# Structural and Functional Principles in Quadriceps Reconstruction

**DOI:** 10.3390/muscles5020041

**Published:** 2026-06-09

**Authors:** Andrei Cretu, Eliza-Maria Bordeanu-Diaconescu, Catalina-Stefania Dumitru, Cristian-Vladimir Vancea, Mihaela-Cristina Andrei, Adriana Serban, Cristian-Sorin Hariga, Cristian-Radu Jecan, Ioan Lascar, Andreea Grosu-Bularda

**Affiliations:** 1 Department 11, Discipline Plastic and Reconstructive Surgery, University of Medicine and Pharmacy Carol Davila, 050474 Bucharest, Romania; andrei.cretu@drd.umfcd.ro (A.C.); andreea.grosu-bularda@umfcd.ro (A.G.-B.); 2 Clinic of Plastic Surgery and Reconstructive Microsurgery, Clinical Emergency Hospital of Bucharest, 014461 Bucharest, Romania; 3 Institute of Doctoral and Postdoctoral Studies, National University of Physical Education and Sports, 060057 Bucharest, Romania

**Keywords:** quadriceps reconstruction, quadriceps muscle, sarcoma, free functional muscle transfer, tendon transfers, quadriceps rehabilitation

## Abstract

Quadriceps muscle and tendon injuries are a significant cause of impairment of the knee extensor mechanism, ranging from minor muscle strains to complete tendon ruptures and extensive defects following oncologic resections. This narrative review provides a comprehensive analysis of contemporary concepts in anatomy, biomechanics, diagnosis, surgical management, and rehabilitation, with a particular focus on reconstructive techniques and functional outcomes. While most muscle injuries respond well to conservative management, complete quadriceps tendon ruptures typically require surgical repair to restore extensor continuity. Both transosseous suture techniques and suture anchor fixation demonstrate reliable outcomes, with no clear superiority in clinical results. Chronic ruptures present additional challenges due to tendon retraction and poor tissue quality, often necessitating advanced reconstruction methods such as V–Y tendon lengthening and augmentation with autografts, allografts, or synthetic materials. In cases of large defects, especially following soft-tissue sarcoma resection, free functional muscle transfer (FFMT) has emerged as a key reconstructive strategy. Common donor muscles include the latissimus dorsi, gracilis, rectus abdominis, and vastus lateralis, each offering specific biomechanical advantages. Functional recovery is strongly influenced by the extent of quadriceps preservation, with better outcomes observed when at least two muscle heads remain functional. Rehabilitation protocols vary depending on the surgical approach. Early controlled mobilisation is generally recommended after tendon repair, whereas FFMT requires a more cautious and prolonged rehabilitation process to allow for flap integration and reinnervation. Overall, optimal outcomes depend on a multidisciplinary approach combining appropriate surgical technique, individualized rehabilitation, and careful patient selection.

## 1. Introduction

The integrity of the quadriceps muscle and tendon is essential for the preservation of knee extension, gait stability, and overall lower limb function. As the primary extensor mechanism of the knee, the quadriceps femoris plays a central biomechanical role in activities ranging from basic ambulation to advanced functional tasks. Disruption of this complex mechanism, whether through traumatic injury, degenerative processes, or oncologic resection, can result in significant functional impairment and reduced quality of life [1,2,3].

Quadriceps injuries encompass a broad clinical spectrum, from minor muscle strains and contusions to complete tendon ruptures and extensive composite defects. While most muscle injuries respond favourably to conservative management, complete quadriceps tendon ruptures typically require surgical intervention to restore the continuity of the extensor mechanism. Chronic ruptures present greater reconstructive challenges due to tendon retraction, degeneration, and compromised tissue quality, often requiring more advanced surgical solutions. Consequently, management requires a comprehensive, multidisciplinary approach integrating expertise in orthopaedics, sports medicine, reconstructive surgery, and rehabilitation. Functional rehabilitation should be initiated early and continued throughout the entire recovery process [4,5,6,7,8,9,10,11,12,13,14,15].

In oncologic settings, wide excision of soft-tissue sarcomas involving the anterior thigh may require partial or total resection of the quadriceps muscle group, leading to severe deficits in knee extension and overall limb function. In these complex cases, reconstructive strategies extend beyond simple tendon repair, involving tendon transfers or free functional muscle transfer to both restore active extension and provide adequate soft-tissue coverage [16].

Over recent decades, significant advances in surgical techniques and rehabilitation protocols have improved functional outcomes even in patients with extensive defects. A wide range of reconstructive options has been described, from primary repair to microsurgical muscle transfers. However, considerable variability persists in clinical practice, particularly regarding optimal technique selection and postoperative rehabilitation strategies. Given the complexity and heterogeneity of quadriceps injuries and their management, this review aims to provide a comprehensive overview of the anatomy, biomechanics, reconstructive options, and rehabilitation principles involved.

Although traumatic quadriceps tendon rupture and oncologic quadriceps loss represent clinically distinct entities, both conditions ultimately compromise the integrity of the knee extensor mechanism and may require complex reconstructive strategies to restore active extension and functional ambulation. This review, therefore, aims to integrate the available evidence regarding anatomy, biomechanics, tendon repair, muscle transfer, and rehabilitation into a unified reconstructive framework centred on restoration of extensor mechanism function.

## 2. Methodology

This study was designed as a narrative review aimed at providing a clinically oriented synthesis of the current literature regarding quadriceps muscle and tendon injuries, with particular emphasis on reconstructive techniques and postoperative rehabilitation strategies. The review addressed both traumatic and oncologic conditions involving the quadriceps extensor mechanism, including acute and chronic tendon ruptures, graft-based reconstructions, tendon transfers, and free functional muscle transfer procedures.

A literature search was conducted using the PubMed, Web of Science, and Google Scholar databases. The search included articles published up to December 2025. Search terms were combined using Boolean operators and included the following keywords and expressions: “quadriceps tendon rupture”, “quadriceps muscle injury”, “extensor mechanism reconstruction”, “quadriceps reconstruction”, “quadriceps tendon repair”, “muscle transfer”, “free functional muscle transfer”, “functional quadriceps reconstruction”, and “rehabilitation after quadriceps repair”.

Studies were initially screened based on title and abstract relevance. Full texts of potentially eligible articles were subsequently reviewed. Preference was given to original clinical studies, systematic reviews, meta-analyses, narrative reviews with significant clinical relevance, and well-documented case series describing surgical techniques, rehabilitation protocols, functional outcomes, and complication profiles. Additional articles were identified through manual screening of the reference lists of selected publications. Inclusion criteria comprised studies addressing the anatomy, biomechanics, diagnosis, surgical management, reconstruction, or rehabilitation of quadriceps muscle and tendon injuries in adult patients. Both traumatic and oncologic aetiologies were considered. Exclusion criteria included articles unrelated to quadriceps reconstruction, studies lacking sufficient surgical or functional outcome data, conference abstracts without full-text availability, isolated technical descriptions without clinical correlation, and non-English language publications. Given the rarity and heterogeneity of extensive quadriceps reconstructions, high-level comparative evidence remains limited, with a substantial proportion of the literature consisting of retrospective series and case reports.

## 3. Anatomy and Biomechanics

The quadriceps femoris muscle is located within the anterior compartment of the thigh and represents the primary extensor mechanism of the knee. It is composed of four principal muscle bellies: the rectus femoris, vastus medialis, vastus lateralis, and vastus intermedius. The rectus femoris occupies the anterior aspect of the thigh, while the vastus medialis and vastus lateralis are positioned medially and laterally, respectively. The vastus intermedius lies deep to the rectus femoris, along the anterior surface of the femur [1].

The vastus lateralis arises from the greater trochanter, gluteal tuberosity, and lateral lip of the linea aspera, whereas the vastus medialis originates from the medial aspect of the femur and medial lip of the linea aspera. The vastus intermedius originates from the proximal anterior and lateral surfaces of the femoral shaft, with some fibres contributing to the articular muscle of the knee by inserting on the suprapatellar recess [2,3,4].

The rectus femoris crosses both the hip and knee joints and originates proximally via three distinct tendinous components. The direct tendon arises from the anterior inferior iliac spine, while the indirect tendon originates from the superior acetabular rim. A smaller reflected tendon inserts on the anterior capsule of the hip joint. Distally, the direct and indirect tendons continue as two aponeurotic laminae extending throughout much of the muscle belly, with the direct tendon forming a superficial lamina and the indirect tendon forming a central sagittal lamina [2,3,4].

Distally, all four muscle bellies converge to form the quadriceps tendon, a thick, multilayered structure that inserts on the superior pole of the patella. The tendon is organised into distinct laminae, with the superficial layer continuous with fibres from the rectus femoris, an intermediate layer receiving contributions from the vastus medialis and vastus lateralis, and a deep layer formed by fibres of the vastus intermedius. A proportion of the superficial fibres extend over the anterior surface of the patella and continue distally as the patellar tendon, inserting on the tibial tuberosity. Additional connective tissue extensions from the vastus medialis and vastus lateralis contribute to the medial and lateral patellar retinacula, further stabilising the extensor mechanism [2,3,4,5].

## 4. Aetiology of Quadriceps Muscle and Tendon Lesions

Acute quadriceps muscle strains and contusions are frequently encountered in athletic settings. Muscle strains most often occur during sudden, forceful eccentric contraction of the quadriceps or from excessive passive stretching or activation of the muscle in a maximally lengthened position. Among the quadriceps muscles, the rectus femoris is the most frequently strained, a predisposition attributed to several factors, such as its biarticular function, high proportion of type II muscle fibres, and complex musculotendinous architecture [6,7].

Quadriceps muscle strains may present with visible contour abnormalities, such as focal bulging or a palpable defect within the muscle belly. Clinical examination should include strength assessment by evaluating resisted knee extension and hip flexion, and palpation along the length of the anterior thigh to identify the point of maximal tenderness and any discontinuity within the muscle [8].

Muscle strains are classically divided into three grades: grade 1, involving minor fibre disruption, mild to moderate pain, minimal or no strength loss, and no palpable defect; grade 2, characterised by greater fibre disruption with significant pain, reduced strength, and sometimes a palpable defect; and grade 3, representing complete muscle rupture with severe pain, complete loss of strength, usually associated with a palpable defect if the examination is performed before haematoma formation [8,9].

Muscle contusions usually occur after direct blows to the muscle and are characterised by pain at the site of injury, localized swelling, limited range of motion, and tenderness to palpation. Clinical examination includes inspection for deformity, swelling, or ecchymosis, palpation to identify tenderness or defects, and strength assessment with resisted knee extension and hip flexion compared with the contralateral side [8,9].

The quadriceps tendon is a robust structure with high tensile strength, connecting the quadriceps muscle group to the patella [10]. Tendon rupture can occur iatrogenically, as a complication after total knee arthroplasty or antegrade tibial nailing, while intra-articular injections carry an increased risk of quadriceps tendon rupture [11,12,13]. Spontaneous rupture of the quadriceps tendon is rare and is usually encountered in patients over 50 years of age, who typically present with acute pain, followed by swelling and tenderness over the distal thigh, skin ecchymosis, a palpable suprapatellar gap, and the inability to extend the knee. In this subset of patients, it is important to exclude a patellar fracture through radiographic examination [12].

Quadriceps tendon rupture may be associated with systemic comorbidities such as gout, rheumatoid disorders, hyperparathyroidism, uraemia, obesity, muscle weakness, age-related tendon degeneration, repetitive microtrauma, and medications such as statins, fluoroquinolones, corticosteroids, and anabolic steroids [13,14]. It can be unilateral or bilateral, with 30% of bilateral simultaneous ruptures occurring in patients with underlying predisposing conditions or repetitive occupational stress [15].

The anterior compartment of the thigh is a common location for soft-tissue sarcomas, and wide tumour excision in this region often requires en bloc resection of the quadriceps femoris muscle, leading to substantial loss of knee extension strength and compromised functional outcomes [16].

## 5. Paraclinical Examination

Conventional radiographs are typically the first-line imaging modality in the acute setting, primarily to exclude osseous involvement such as patellar fractures, avulsion fractures of the superior pole of the patella, or associated femoral fractures. In cases of quadriceps tendon rupture, indirect radiographic signs may include an inferior riding patella (patella baja), soft tissue swelling, and obliteration of the suprapatellar pouch. Radiographs are also useful in the delayed setting in identifying myositis ossificans, a complication of severe muscle contusions characterized by heterotopic bone formation [13,17,18,19].

Musculoskeletal ultrasound is a rapid, cost-effective, and dynamic imaging modality that allows real-time evaluation of the quadriceps muscle and tendon. It is particularly useful in the assessment of acute muscle injuries, enabling identification of fibre disruption, intramuscular haematomas, and partial or complete tendon tears. In muscle strains, ultrasound may demonstrate hypoechoic areas corresponding to oedema or hemorrhage, as well as disruption of the normal fibrillar architecture. In cases of quadriceps tendon rupture, discontinuity of the tendon fibres, retraction of the proximal stump, and the presence of a hypoechoic haematoma can be visualized. Dynamic examination during passive or active movement may further aid in differentiating partial from complete tears. However, ultrasonography is operator-dependent and may be limited in evaluating deep structures such as the vastus intermedius or in patients with significant soft-tissue swelling [17,20,21,22].

Magnetic resonance imaging (MRI) is considered the gold standard for the evaluation of quadriceps muscle and tendon injuries due to its superior soft-tissue contrast and multiplanar capability [23]. MRI provides detailed information regarding the presence of oedema and haemorrhage, the location, extent, and severity of muscle strains, as well as the precise characterization of tendon lesions. The extent of fibre disruption and involvement of the myotendinous junction can be accurately assessed, allowing reliable grading of muscle injuries. In quadriceps tendon ruptures, MRI demonstrates discontinuity of tendon fibres, retraction, and associated fluid collections. MRI is also essential in chronic cases, where it can identify tendon degeneration, scar tissue formation, fatty infiltration, and the degree of tendon retraction, all of which are critical factors in determining the feasibility of primary repair [24,25,26]. In patients with suspected or confirmed soft-tissue sarcomas of the anterior thigh, MRI remains the imaging modality of choice for local staging, allowing accurate delineation of tumour extent, involvement of adjacent neurovascular structures, and planning of surgical margins [27].

Computed tomography can be used in the assessment of complex fractures, evaluation of osseous involvement in tumour cases, preoperative planning when bone reconstruction is required, and in analysing the morphology of the quadriceps tendon and its patellar insertion [28].

In selected cases, particularly in chronic injuries or following reconstruction, electromyography may be used to evaluate muscle viability and monitor reinnervation of transferred muscles. This can provide valuable prognostic information regarding functional recovery [29,30].

Laboratory investigations are generally not required for isolated traumatic injuries but may be indicated in cases of suspected systemic disease predisposing to tendon rupture, such as renal failure, endocrine disorders, or inflammatory arthropathies [31].

## 6. Treatment

### 6.1. Quadriceps Muscle Lesions

Surgical management of muscle strains and contusions should be approached with caution, as well-conducted conservative treatment usually leads to favourable outcomes, supporting the principle that most muscle injuries respond well to non-operative treatment. Nevertheless, surgery may be considered in the cases of large intramuscular haematomas, complete muscle ruptures in the absence of effective agonist muscles, partial tears involving more than 50% of the muscle belly, or persistent extension-related pain lasting longer than 4–6 months after injury. Surgical intervention may be particularly indicated when persistent pain is associated with a functional extension deficit, suggesting restrictive scar adhesions requiring operative release [32].

### 6.2. Quadriceps Tendon Rupture

Partial ruptures are the most common presentation and are typically managed non-operatively, often with immobilisation in a cylinder cast [13].

In complete quadriceps tendon ruptures at or near the osteotendinous junction without significant tissue loss, primary tendon-to-bone repair remains the treatment of choice. For tears located more proximally, a direct end-to-end suture is generally preferred in the acute setting [17].

For ruptures near the patella, transosseous suture techniques with fixation through patellar drill holes have been employed for decades and are considered the gold standard [33,34]. Suture anchor repair represents a more recent alternative, offering fixation without the need for transosseous drilling.

Studies have demonstrated the biomechanical superiority of suture anchors over transosseous suture techniques, potentially allowing early functional rehabilitation, resulting in earlier rehabilitation and improved functional recovery [35,36].

In contrast, Plesser et al. found no significant differences in clinical outcomes or rerupture rates between transosseous suture repair and suture anchor fixation, with patients demonstrating good results following both techniques. The transosseous suture technique consisted of drilling three to four longitudinal tunnels through the patella and suturing the tendon to the patella using either absorbable or non-absorbable sutures; suture patterns used included the Mason-Allen technique, Krackow technique, and Kessler–Kirchmayr technique. Suture anchor repair consisted of drilling three pilot holes in the proximal pole of the patella with a 3.2 mm drill bit, followed by placement of three 5.5 mm titanium corkscrew suture anchors armed with two strands of high-strength non-absorbable suture and fixation of the tendon to the patella using a modified Mason-Allen stitch pattern [37].

In a study by Bushnell et al., the authors suggested that the use of suture anchors avoids trauma to the patella and patellar tendon, dissection at the apex of the patella, and the placement of non-absorbable knots at the apex of the patella, while also reducing surgical time [38].

In chronic ruptures, the three-layered structure of the quadriceps tendon progressively deteriorates as the tendon retracts, collagen fibres shorten, and scar tissue develops. When diagnosis and treatment are delayed, these changes often result in a significant tendon gap, making primary tendon-to-bone repair difficult or impossible. In these cases, reconstruction of the quadriceps tendon often requires V–Y tendon lengthening techniques and reconstruction using autograft or allograft tissue [39,40,41].

The Scuderi technique may be employed either as a primary operative method or as a secondary reinforcement procedure. The procedure involves a direct repair of the quadriceps tendon rupture, augmented by a partial-thickness triangular tendon flap that is turned down and sutured over the anterior surface of the patella to reinforce the repair [42].

The Codivilla technique involves lengthening of the quadriceps tendon through a full-thickness inverted V–Y plasty. A V-shaped incision is created in the tendon, allowing distal advancement of the triangular tendon segment to facilitate reapproximation to the patella. The proximal limbs of the incision are then closed side-to-side, and the remaining tendon flap can be turned down to provide additional reinforcement. Fixation is commonly augmented with suture anchors placed in the patella, enhancing tendon-to-bone stability and overall construct strength [40].

In a study by Mahoney et al., the authors advocated the use of mesh augmentation using LARS (ligament augmentation and reconstruction system) in addition to a Codivilla V–Y plasty to extend the tendon. The LARS band is composed of synthetic materials and avoids the temporary loss of strength associated with biological graft remodelling, while reducing the risk of postoperative lengthening during the early healing period. In these cases, the LARS acted as a protective splint, allowing early rehabilitation without increasing the risk of repair failure [43].

Commonly used autografts for quadriceps tendon defects include the semitendinosus and gracilis tendons, either individually or in combination, owing to their adequate length, favourable biomechanical properties, and relatively low donor-site morbidity [44,45,46]. Fascia lata autografts may be employed when broader tissue coverage or augmentation is required, especially in the presence of large tendon defects, and may be used to recreate the anterior retinaculum of the knee in conjunction with other autografts [47].

In the event of autograft failure, an Achilles tendon allograft may be considered as an alternative for revision reconstruction. The advantages of Achilles tendon allografts, including the absence of donor-site morbidity and the elimination of tendon harvesting, must be carefully balanced against their disadvantages, which include non-physiological stiffness, risk of infection or disease transmission, limited availability in some countries, and increased cost [48,49].

Synthetic grafts, typically composed of polymer-based materials, offer high intrinsic tensile strength, providing sufficient strength to withstand physiological loading. Various synthetic options have been described in the literature, including polypropylene meshes such as Marlex, the Leeds–Keio ligament constructed from braided polyester, and the Neoligaments Poly-Tape system made of polyethylene terephthalate. In a study by Permutt et al., a combined reconstructive approach was described, consisting of mobilisation of the proximal quadriceps tendon and muscle belly using a V–Y tendon plasty, advancement of the tendon, and the Krakow technique through intraosseous patellar tunnels, augmented with Poly-Tape and an Achilles’ tendon allograft [50,51,52,53].

Coladonato et al. reported that advanced age, higher body mass index, female sex, retinacular involvement, and active smoking were significantly associated with poorer outcomes, including reduced range of motion, postoperative extensor lag, and increased complication rates [54]. A systematic review by Ciriello et al. reported less favourable outcomes following delayed repair of the quadriceps tendon [55].

### 6.3. Large Defects

Following excision of a soft-tissue sarcoma, total or partial reconstruction of the quadriceps using a free functional muscle transfer is performed to reduce functional deficits associated with loss of the thigh extensor compartment and to reconstruct the resulting soft-tissue defect after excision [16].

The most common flaps used in quadriceps reconstruction are the latissimus dorsi, gracilis, chimeric anterolateral thigh flaps, tensor fascia lata, rectus abdominis, and rectus femoris [56,57,58].

Selection of a donor muscle for functional transfer requires adequate contractile strength, sufficient excursion, and an adaptable shape suited to the recipient site. As muscle force is directly related to cross-sectional area, bulkier muscles can generate greater power. The latissimus dorsi, the largest myocutaneous flap in the body, offers a cross-sectional area comparable to the rectus femoris and substantially greater bulk than the gracilis or tensor fasciae latae, making it well suited for powerful functional reconstruction. Strap muscles, such as the latissimus dorsi and gracilis, provide greater excursion during contraction compared with pennate muscles, including the rectus femoris. Due to its limited size, the gracilis is better suited for smaller defects. In contrast, the rectus femoris provides substantial muscle mass but is associated with donor-site morbidity and reduced knee extension strength, and its pennate architecture results in less excursion compared with the latissimus dorsi. Although the rectus abdominis is a large and well-innervated muscle, its transfer as a functional unit is technically challenging due to its segmental innervation [59,60].

To optimize outcomes, the preoperative criteria include preserved joint mobility, intact sensation, adequate gliding surfaces, sufficient soft-tissue coverage, and the presence of an expendable, uncompromised neurovascular bundle at the recipient site suitable for anastomosis to the pedicle of the transferred muscle [59].

Across multiple studies, preservation of at least two quadriceps components consistently appears to be one of the strongest predictors of satisfactory postoperative knee extension strength and functional ambulation and should therefore play a central role in reconstructive planning.

#### 6.3.1. Latissimus Dorsi

The patient is placed in a lateral decubitus position, and the flap is harvested from the same side as the tumour to facilitate simultaneous excision and flap harvest and thus reduce the operative time [59]. For reconstruction with free functional latissimus dorsi following oncologic excision, a myocutaneous latissimus dorsi flap is harvested, including its humeral tendon insertion, with the muscle dissected along its full length, while carefully preserving the thoracodorsal nerve [60,61]. The native resting tension of the muscle is marked using sutures placed at regular distances before detachment, usually at 5 cm intervals [59,60,61].

The extent of quadriceps resection is determined by tumour location, with preservation of the descending branch of the lateral circumflex femoral vessels and motor branches of the femoral nerve whenever possible [60]. The sartorius muscle is typically preserved following tumour resection, serving initially to protect and cover the femoral vessels. It can subsequently be incorporated into the reconstruction by transfer to the extensor mechanism. When the entire quadriceps muscle group is resected, the femur represents the deep surgical margin, which is why a subperiosteal resection is performed [59].

The free functional latissimus dorsi is inset between the proximal quadriceps remnant and the patellar tendon, or to the rectus femoris tendon when all quadriceps heads have been resected, with resting tension adjusted using the pre-marked sutures to ensure stable fixation and optimal force transmission [59]. Microvascular anastomosis is performed between the subscapular and lateral circumflex femoral vessels, followed by nerve coaptation close to the muscle to reduce denervation time [60].

Innocenti et al. first described the inset with the proximal tendon of the latissimus dorsi sutured to the proximal remaining quadriceps stump when available or secured directly to the anterior surface of the proximal femur using titanium suture anchors, while the distal muscular portion of the flap is secured to the distal quadriceps tendon [59]. Later, Muramatsu et al. and Houdek et al. described an alternative inset configuration, with the tendinous portion of the latissimus dorsi secured to the distal ends of the quadriceps at the level of their tendinous insertions and the muscular portion of the latissimus either directly sutured to the proximal quadriceps or fixed to the femur or ischium using suture anchors [60,61].

The sartorius muscle, sometimes preserved following tumour resection, can be mobilised and released distally, then rerouted and sutured to the quadriceps tendon to augment knee extension strength [59], although some authors prefer not to supplement the reconstruction with the tendon transfer [61]. A segment of fascia lata can be harvested from the ipsilateral limb and subsequently sutured over the junction between the latissimus dorsi muscle and the quadriceps tendon, reinforcing the muscle-tendon interface and enhancing the strength of the repair [59].

#### 6.3.2. Rectus Abdominis

Surgically, the anterior rectus sheath is incised laterally to expose the lateral border of the rectus muscle. The segmental intercostal nerves are carefully identified, dissected, and isolated. A handheld nerve stimulator is used to determine the dominant motor branches that produce the strongest muscle contractions. At the recipient site, the number of available femoral nerve branches in the thigh is assessed, and a corresponding number of dominant motor nerves is selected and dissected within the rectus muscle [62].

The linea semilunaris is sharply divided using diathermy on either side of the selected nerve. A loose areolar tunnel is then developed around the nerve within the neurovascular plane of the abdominal wall, between the transversus abdominis and internal oblique muscles. This technique reliably allows harvesting approximately 10–12 cm of nerve length [62].

The tendinous intersections of the rectus abdominis muscle are secured under appropriate tension using large, non-absorbable sutures, anchoring them proximally and distally to the residual tendons of the quadriceps mechanism. The skin paddle is inset with a standard three-layer closure. It is important to note that the TRAM skin island is highly mobile and can be rotated up to 90° when necessary, allowing alignment with the orientation of the underlying muscle to optimize reconstruction [62].

#### 6.3.3. Gracilis

The gracilis muscle is harvested in its entirety, with meticulous dissection of the proximal origin directly from the ischial periosteum and release of the distal tendon at the level of the pes anserinus [63].

When performing an ipsilateral pedicled gracilis transfer, the vascular pedicle is carefully dissected proximally to its origin from the profunda femoris vessels beneath the adductor longus. A tunnel is then developed deep to the adductor longus and superficial to the adductor brevis and magnus. If necessary, this passage is extended by creating a controlled slit along the superior border of the adductor longus to allow sufficient length for transposition of the gracilis from its native medial position to a new lateral position [63].

Meticulous attention is paid to avoid compression, torsion, or kinking of the vascular pedicle, which must curve superiorly and laterally over the profunda vessels as the muscle is transferred. Once adequately mobilised, the gracilis is positioned in the rectus femoris location to restore functional knee extension [63].

The motor branch of the obturator nerve to the gracilis can be divided and prepared for nerve transfer. Neurorrhaphy is then performed to the most suitable available motor branch of the femoral nerve (preferably the residual motor branch to the rectus femoris) to enable functional reinnervation [63].

#### 6.3.4. Vastus Lateralis

The vastus lateralis (VL) is the largest and most powerful component of the quadriceps muscle group. Its substantial bulk allows it to effectively obliterate large defects, and it possesses a long, reliable neurovascular pedicle that facilitates straightforward microvascular anastomosis and nerve coaptation, often permitting a “like-for-like” reconstruction with excellent vessel and axonal calibre match [56,57].

A skin paddle is designed over the central paramedian thigh, guided by Doppler ultrasound identification of a suitable perforator. The main vascular pedicle is located in the intermuscular plane between the vastus lateralis and rectus femoris and is traced proximally. When harvesting the vastus lateralis, distal circumferential dissection is performed with meticulous care to preserve the native extensor mechanism. Resting tension of the tendinous portion can be assessed by marking 5 cm reference intervals with sutures [57].

The distal tendon is then transected, and the muscle is reflected proximally to expose the motor branch to the vastus lateralis and the descending branch of the lateral circumflex femoral artery. Once the motor nerve and vascular pedicle are clearly identified, the proximal vessels of the descending branch are divided, and the flap is transferred to the contralateral thigh [57].

Under microscopic visualisation, the descending branch of the lateral circumflex femoral artery in the recipient site is anastomosed to the corresponding pedicle of the free flap. The motor branch to the vastus lateralis is trimmed back to healthy fascicles before being coapted to the recipient motor nerve [57].

The proximal muscular fascia is secured to the remnant vastus lateralis. To confirm minimal tension across the vascular pedicle during distal traction, the underlying tendon is first anchored proximally to the native vastus lateralis tendon. Finally, a stitch is placed in the vastus lateralis tendon to secure it firmly to the extensor mechanism [57].

For incorporation of the crural fascia, a subfascial dissection is first carried out to identify the perforating branches arising from the descending branch of the lateral circumflex femoral artery. Once adequate perforator perfusion is confirmed, a laterally extended rectangular segment of crural fascia supplied directly by these perforators is carefully delineated and incised. The harvested fascial segment is then folded onto itself to increase structural strength and secured with non-absorbable sutures to create a robust construct suitable for tendon or extensor mechanism reinforcement [57].

#### 6.3.5. Tendon Transfers

An alternative to total functional quadriceps resection with free muscle transfer is local tendon transfer using residual thigh flexors. This approach may yield comparable long-term outcomes and, in some cases, superior short-term functional recovery.

Pritsch et al. reviewed 15 patients who underwent extensive quadriceps resection followed by reconstruction with pedicled muscle transfers, including the sartorius, biceps femoris, and semitendinosus. Functional assessment using the MTS score demonstrated good to excellent results in 13 patients and fair results in two, leading the authors to advocate the transfer of the remaining musculature as a reliable method for restoring the knee extensor mechanism [64].

Similarly, Steinau et al. proposed distal detachment and anterior transposition of the biceps femoris tendon to function as a knee extensor. Patellar stability can be further enhanced through adjunctive transfer of the gracilis muscle to improve medial patellar alignment [65].

While tendon transfer represents a valuable reconstructive option—particularly when two major quadriceps heads are preserved—its utility becomes limited in cases involving resection of three or all four components. In such scenarios, tendon transfer alone cannot provide adequate soft-tissue coverage. Additionally, the creation of supplementary surgical planes may theoretically increase the risk of local tumour dissemination due to further manipulation of tissues in proximity to the oncologic field [60].

A comparision of reconstructive techniques for quadriceps reconstruction is depicted in Table 1.

## 7. Rehabilitation

Rehabilitation after quadriceps tendon repair generally favours early controlled mobilisation combined with protective bracing, rather than prolonged immobilisation. A hinged knee brace is typically applied immediately postoperatively, allowing controlled motion while protecting the repair. Complete immobilisation is reserved only for specific situations, such as fragile repairs, compromised soft-tissue or impaired wound healing, chronic ruptures, or revision cases. Early motion is usually initiated within the first postoperative week, beginning with a range of motion from full extension to approximately 45° of flexion. During the first two weeks, flexion is performed actively while extension remains passive to minimize strain on the repair. Range of motion is then gradually increased by about 15° per week until full mobility is achieved. Muscle activation starts immediately, with isometric quadriceps and hamstring exercises introduced on the first postoperative day. Full weight-bearing is often permitted with crutches, provided the knee is locked in full extension for approximately six weeks. Active knee extension exercises are typically initiated at six weeks, and both the brace and crutches are discontinued around 12 weeks once adequate quadriceps strength has been restored [66].

A structured and slightly more conservative progression is described by Portugal et al., in which formal physical therapy begins at two weeks postoperatively [69]. At that stage, gentle range-of-motion exercises are introduced, consisting of active knee flexion and passive extension. For the first six weeks, patients use a hinged knee orthosis locked in full extension during ambulation to protect the repair. Rehabilitation progresses from six weeks onward with the introduction of active knee extension and gradual dynamic quadriceps strengthening. Full weight-bearing is typically achieved around seven weeks, reflecting satisfactory tendon healing. Between 10 and 12 weeks, the brace is progressively unlocked to allow full flexion and is subsequently discontinued. Aquatic therapy is introduced at approximately 12 weeks to facilitate low-impact strengthening and mobility. Functional recovery continues over several months, with return to work generally expected around six months postoperatively [67,68].

Another rehabilitation protocol described by Geyer et al. emphasizes graded increases in both range of motion and weight-bearing under stricter protection during the early phase. In this approach, the knee is immobilised in a hinged brace for six weeks, with staged increases in permitted flexion: up to 30° during weeks 1–2, 60° during weeks 3–4, and 90° during weeks 5–6. Weight-bearing is initially restricted to approximately 20 kg and allowed only with the knee locked in full extension. Full weight-bearing is gradually introduced beginning in the seventh postoperative week. Rehabilitation starts immediately after surgery, focusing on passive knee flexion within the allowed limits and isometric quadriceps activation performed in full extension to maintain muscle engagement without compromising the repair. From week seven onward, active knee extension exercises are incorporated, marking the transition to more dynamic strengthening. Regular physiotherapy sessions, typically two to three times per week, support consistent functional recovery [69].

Rehabilitation following free functional muscle transfer for quadriceps reconstruction is characterized by a more cautious and prolonged progression, reflecting the need to protect the transferred muscle, ensure vascular integration, and allow time for reinnervation. In the early postoperative phase, rehabilitation is limited, and mobilization out of bed is typically delayed until after approximately 21 days. During this period, the focus is on general conditioning, including postural exercises, trunk control, and upper limb strengthening, while mobility is restricted. Loading of the operated limb is avoided or introduced very cautiously, with gradual progression only after the first month postoperatively. As rehabilitation progresses, weight-bearing activities are introduced gradually, often requiring the continued use of a brace to protect the reconstruction. Compared to other reconstructive techniques, FFMT patients progress more slowly in ambulation, with progressive loading carefully monitored to avoid compromising the flap as depicted in Table 2 [70].

## 8. Discussion

Despite the large number of described reconstructive techniques, high-level comparative evidence remains limited. Most available studies consist of retrospective case series and technical reports, making direct comparison between reconstructive strategies difficult. Consequently, surgical decision-making is largely guided by defect size, residual quadriceps preservation, soft-tissue requirements, and surgeon experience rather than standardized evidence-based algorithms.

Quadriceps tendon rupture represents a severe disruption of the knee extensor mechanism, resulting in significant functional impairment and loss of active knee extension [10]. Accurate differentiation between partial and complete ruptures is essential, as it directly influences treatment strategy. Management is largely dictated by the extent of the tear. Partial ruptures, particularly those with preserved extensor function, can often be managed non-operatively with immobilisation followed by structured rehabilitation [13]. In contrast, complete ruptures require surgical repair to restore the continuity of the extensor mechanism [17]. In the acute setting, primary repair yields the most favourable outcomes, with both transosseous suture techniques and suture anchor fixation demonstrating reliable results. Although biomechanical studies suggest superior initial fixation strength with suture anchors, clinical outcomes appear comparable between techniques, indicating that surgical expertise and appropriate tensioning may be more critical determinants of success than the fixation method itself [33,34,35,36,37]. Chronic quadriceps tendon ruptures present a greater reconstructive challenge due to tendon retraction, scar formation, and poor tissue quality. In such cases, primary repair is often not feasible, and advanced reconstructive techniques are required. V–Y tendon lengthening procedures, such as the Codivilla technique, allow distal advancement of the retracted tendon, while augmentation with autografts, allografts, or synthetic materials may be necessary to bridge large defects and reinforce the repair [39,40,41].

The quadriceps femoris muscle plays a central role in lower limb biomechanics, acting as the primary extensor of the knee and a key contributor to gait, posture, and functional mobility. Among its components, the vastus lateralis provides the greatest contribution to extension strength, followed by the vastus medialis and rectus femoris, the latter serving as both a knee extensor and hip flexor. This functional hierarchy has important implications in both injury patterns and reconstructive strategies [60].

A consistent finding across multiple studies is that functional outcomes correlate strongly with the number of preserved quadriceps heads. Muramatsu et al. observed that isolated resection of only one muscle of the group did not result in substantial impairment of knee extension. Patients who underwent resection of two major quadriceps heads retained moderately preserved extension strength even before reinnervation of the transferred muscle. In contrast, resection of three or more components of the quadriceps led to a marked reduction in extension power, with meaningful functional recovery occurring only after successful reinnervation of the muscle transfer [60].

Several studies have demonstrated a direct relationship between the extent of quadriceps resection and postoperative muscle strength. Innocenti et al. reported a mean postoperative MMT score of 3 (range 2–4), with poorer outcomes observed in patients with fewer preserved quadriceps heads, while Hood et al. reported a mean MMT of 3.5 ± 1 [45,61]. Muramatsu et al. further demonstrated that complete quadriceps resection resulted in poor initial strength with only partial long-term recovery, whereas resection of two heads yielded near-normal functional outcomes. Importantly, functional recovery after three-head resections varied according to the preserved component, suggesting that preservation of the vastus medialis may play a particularly important role in restoration of effective knee extension [60].

The latissimus dorsi remains one of the most frequently used donor muscles for functional quadriceps reconstruction because of its reliable vascular anatomy, substantial excursion, and favourable functional capacity. However, its mass corresponds to only approximately one-third of the native quadriceps, limiting its ability to fully restore knee extension after complete quadriceps excision. Functional outcomes are therefore substantially better when at least one or two native quadriceps heads can be preserved [59,60,61]. Reduced excursion of the transferred muscle may occur following extensive anterior thigh resection with periosteal stripping due to adhesions between the muscle and femoral cortex; in these cases, fascia lata coverage may improve the gliding surface and preserve mobility [59,60,61]. When combined with the sartorius transfer, the latissimus dorsi contributes significantly to knee stability and provides sufficient active extension to enable ambulation without external support, even in patients with total quadriceps loss [59]. Although the latissimus dorsi can provide sufficient active extension to restore ambulation, complication rates remain considerable, particularly in irradiated patients undergoing periosteal stripping, where femoral fractures represent a major concern [59,61]. To prevent this, prophylactic femoral stabilisation has been advocated, reducing the radiation-associated femoral fractures to zero, as reported in a study by Houdek et al. [61,72]. Donor-site morbidity is generally limited, with only subtle long-term shoulder dysfunction reported in most patients [61].

Although traditionally considered suboptimal for functional reconstruction because of its segmental innervation, the rectus abdominis has demonstrated satisfactory functional recovery following reinnervation in selected patients [62,72]. The presence of dominant motor branches and overlapping neural input appears sufficient to permit effective muscle contraction after transfer. Grinsell et al. reported good functional outcomes following TRAM and VRAM flap reconstruction, with postoperative MRC power scores ranging from 3 to 5 [62]. A major advantage of the rectus abdominis is the ability to provide substantial soft-tissue coverage in addition to functional reconstruction, particularly in extensive oncologic defects. The choice between a transverse (TRAM) and vertical (VRAM) skin paddle depends on the size of the cutaneous defect and the volume of dead space requiring obliteration, with larger defects generally necessitating a transverse skin island. Donor-site morbidity, such as abdominal wall hernia (0–10% of cases) or abdominal bulge (<15% of cases), remains acceptable and may be further reduced with mesh reinforcement of the abdominal wall [62,73].

The gracilis represents a technically straightforward reconstructive option, particularly when used as a pedicled transfer. Because it lies within the same operative field as the tumour resection, donor-site morbidity is minimal, and microsurgical anastomosis may be avoided [63]. Its long distal tendon and robust fascial attachments facilitate secure fixation within the extensor mechanism, while preservation or shortening of the obturator motor branch may optimize reinnervation depending on the level of femoral nerve resection. The decision to use a free or pedicled gracilis flap depends primarily on the extent of the associated cutaneous defect. The gracilis is particularly well suited for smaller or moderate defects, whereas larger cutaneous defects may require additional free flap coverage, such as an anterolateral thigh (ALT) or deep inferior epigastric perforator (DIEP) flap. Compared with larger donor muscles such as the latissimus dorsi or rectus abdominis, functional donor-site morbidity following gracilis harvest appears limited [63]. In addition, when used as a pedicled transfer, concerns related to recipient vessel selection are eliminated—particularly in a previously irradiated or vessel-depleted field, where vein grafts may be otherwise required [63].

Transfer of the contralateral vastus lateralis provides a biomechanically attractive reconstructive option because it closely reproduces the native line of pull, fibre orientation, and excursion of the quadriceps mechanism, especially since it is the largest and most powerful component of the quadriceps femoris [57]. Preservation of the remaining quadriceps heads at the donor site generally maintains adequate residual knee extension strength and limits donor morbidity. In addition, incorporation of fascia lata, iliotibial tract, or tensor fasciae latae components may further assist tendon reconstruction, knee stabilisation, and soft-tissue coverage in extensive defects [56,57,74]. Nevertheless, complete restoration of physiologic quadriceps function remains difficult in the absence of the native rectus femoris and vastus medialis, emphasizing the importance of residual muscle preservation for optimal long-term recovery [57].

The main free functional muscle transfer (FFMT) techniques available for quadriceps reconstruction are summarized in Table 3.

Tendon transfer techniques remain valuable reconstructive options in selected patients; however, their utility becomes limited in sarcomas involving both anterior and posterior compartments, where hamstring musculature may also require resection for oncologic clearance [61]. Furthermore, pedicled hamstring transfers have been associated with a substantial reduction in knee flexion strength, reported between 28% and 67%, requiring careful balance between restoration of knee extension and preservation of residual flexion function [75].

**Figure 1:**
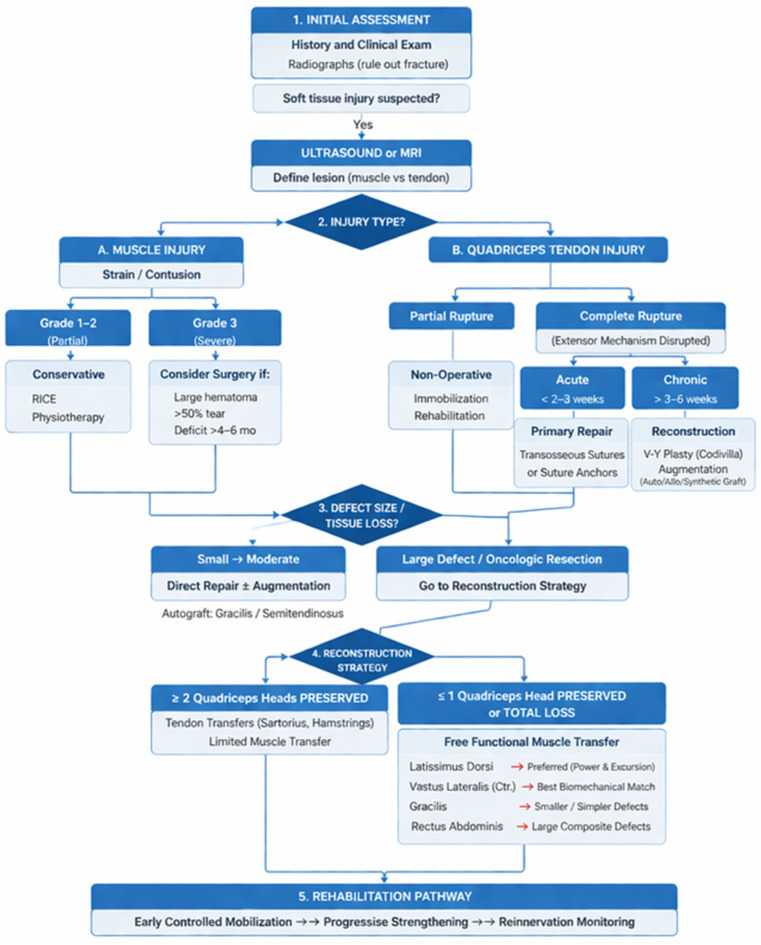
Decision-making algorithm in the management of quadriceps extensor mechanism injuries [13,16,17,23,32,33,37,40,44,50,57,59,60,61,62,63,64].

Given the heterogeneity of quadriceps injuries and reconstructive scenarios, treatment selection should be individualized according to injury chronicity, defect size, residual quadriceps preservation, soft-tissue requirements, and patient functional demands. Based on the currently available literature and reconstructive principles discussed in this review, Figure 1 proposes a practical clinical decision-making algorithm, intended to guide treatment selection across traumatic and oncologic quadriceps defects.

The algorithm emphasizes several key reconstructive principles consistently identified across the literature, including the importance of early repair in acute tendon rupture, the role of residual quadriceps preservation in predicting functional recovery, and the need for free functional muscle transfer in extensive composite defects with loss of multiple quadriceps components. Although not intended as a formal guideline, the proposed framework may assist clinical decision-making in complex reconstructive scenarios.

Skeletal muscle regeneration following injury and reconstruction is influenced by several molecular pathways, among which mTOR signalling plays a central role in protein synthesis, hypertrophy, regeneration, and reinnervation. Recent evidence suggests that modulation of anabolic signalling pathways may influence functional recovery following muscle transfer and rehabilitation. Although current treatment strategies remain primarily surgical, biologic augmentation targeting muscle regeneration pathways may represent an important future direction in quadriceps reconstruction [77,78].

From a clinical perspective, preservation of adequate nutritional status, progressive mechanical loading, and structured rehabilitation may partially exert their beneficial effects through modulation of anabolic signalling pathways, including mTOR. Although current reconstructive strategies remain primarily surgical, future advances in biologic augmentation and pharmacologic modulation of muscle regeneration pathways may further improve functional outcomes following quadriceps reconstruction [77,78].

## 9. Limitations

This review has several limitations. First, as a narrative review, the study was not designed as a systematic review or meta-analysis, and therefore, formal risk-of-bias assessment and quantitative evidence synthesis were not performed. Second, the available literature on extensive quadriceps reconstruction remains heterogeneous and consists predominantly of retrospective case series, technical reports, and small patient cohorts. Direct comparison between reconstructive strategies is therefore limited. Third, variability in rehabilitation protocols, outcome measures, and follow-up duration across studies complicates the interpretation of functional results. Finally, many reconstructive techniques are described only in highly specialized microsurgical centres, which may limit the generalizability of reported outcomes. In addition, the predominance of low-level evidence in the reconstructive literature limits the ability to establish definitive superiority between competing surgical techniques.

## 10. Conclusions

Quadriceps muscle and tendon injuries represent a complex clinical entity with significant functional implications, particularly when the integrity of the extensor mechanism is compromised. Management strategies must be individualized, considering injury type, defect size, and the extent of preserved musculature. While primary repair remains the cornerstone of treatment in acute tendon ruptures, chronic lesions and large composite defects often require advanced reconstructive approaches, including graft augmentation, tendon transfers, or free functional muscle transfer.

Functional outcomes are closely related to the degree of quadriceps preservation and the suitability of the selected reconstructive technique. Advances in microsurgical reconstruction and rehabilitation have expanded the possibilities for restoring knee extension and ambulation even in extensive defects. However, optimal results depend on a multidisciplinary approach that integrates precise surgical execution with tailored rehabilitation protocols. Future efforts should focus on standardizing treatment algorithms and refining reconstructive strategies to further improve functional recovery and patient quality of life.

## Figures and Tables

**Table 1:** Comparison of reconstructive techniques for quadriceps reconstruction [57,59,60,61,62,63,64,65,66,67,68,69,70,71,72,73,74,75]. FFMT = free functional muscle transfer.

Technique	Main Indications	Advantages	Limitations	Donor-Site Morbidity	Functional Outcomes
**Primary tendon repair**	Acute complete tendon rupture	Simple, reliable, preserves native anatomy	Limited utility in chronic rupture	Minimal	Excellent when performed early
**V–Y plasty (Codivilla)**	Chronic rupture with tendon retraction	Allows distal advancement	Limited in massive defects	Minimal	Good when tissue quality adequate
**Autograft reconstruction *(gracilis/semitendinosus/fascia lata)***	Moderate tendon defects	Biologic integration, lower cost	Donor-site morbidity, graft size limitations	Low–moderate	Generally satisfactory
**Allograft reconstruction**	Large defects/revision cases	No donor morbidity	Cost, availability, infection risk	None	Variable
**Synthetic augmentation**	Poor tissue quality/reinforcement	High tensile strength	Infection, stiffness, long-term durability concerns	None	Variable
**Tendon transfer**	≥2 preserved quadriceps heads	Avoids FFMT in selected cases	Reduced flexion strength	Moderate	Good in selected patients
**Latissimus dorsi FFMT**	Extensive quadriceps loss	Powerful muscle, long excursion	Microsurgery required, fracture risk	Mild shoulder dysfunction	Good ambulation recovery
**Gracilis FFMT**	Smaller defects	Minimal donor morbidity	Limited muscle bulk	Minimal	Moderate
**Rectus abdominis FFMT**	Large composite defects	Excellent soft-tissue coverage	Abdominal wall morbidity	Moderate	Good in selected cases
**Vastus lateralis transfer**	Biomechanic match	Native line of pull	Contralateral donor sacrifice	Low	Good functional restoration

**Table 2:** Comparison of postoperative rehabilitation protocols; [66,67,68,69,70]. ROM = range of motion, WB = weight bearing.

Parameter	Acute Tendon Repair (Primary Repair)	Chronic Reconstruction (Augmentation/Grafts)	Free Functional Muscle Transfer Reconstruction)
**Immobilization**	Hinged knee brace in extension for ~6 weeks	Longer protection, brace locked in extension for 6–8 weeks	Prolonged protection, brace in extension for 8–12 weeks
**ROM progression**	Early controlled ROM (0–40° in first 2 weeks), progress to full by 6–8 weeks	Slower progression, full ROM by 10–12 weeks	Passive ROM after 6 weeks, active-assisted after 10–12 weeks, full ROM by 16–20 weeks
**Weight-bearing**	Early protected WB with brace, progress to full by 6 weeks	Partial WB at 6 weeks, full WB by 10–12 weeks	Delayed; partial WB after 10–12 weeks, full WB after 14–16 weeks
**Active extension**	Begin around 6 weeks	Begin after 8–10 weeks based on healing	After signs of reinnervation (usually 3–6 months)
**Strengthening**	Isometric early, progressive after 8–10 weeks	Progressive after 12 weeks	Gradual strengthening once active contraction is obtained
**Return to activity/sport**	4–6 months (low-impact), 6–9 months (higher demand)	6–9 months (low-impact), 9–12 months (higher demand)	10–18 months (according to reinnervation and strength recovery)

**Table 3:** Comparative overview of free functional muscle transfers for quadriceps reconstruction [57,59,60,61,62,63,71,72,73,74,76].

Donor Muscle (FFMT Option)	Advantages	Disadvantages/Limitations	Indications	Microsurgical Anastomosis Required	Donor-Site Morbidity	Expected Functional Recovery	Key Complications
**Latissimus dorsi**	Powerful muscle with long excursion Reliable anatomy and pedicle length Thin, well-vascularized muscle Good track record in knee extensor reconstruction	Requires patient repositioning (prone) Bulky muscle may need debulking Shoulder weakness (rarer with tendon sparing) Longer operative time	Extensive quadriceps loss Chronic defects after trauma or revision surgery Failed previous reconstructions	Yes	Mild to moderate (shoulder weakness, seroma, scapular winging rarely)	Good to excellent active knee extension in most series; ambulation commonly achieved	Partial flap loss/necrosis Infection Shoulder weakness Donor-site seroma Scar problems
**Rectus abdominis**	Strong muscle with straight line of pull Large muscle volume Reliable pedicle (superior epigastric vessels) Good for composite tendon defects	Abdominal wall weakness Risk of hernia/bulge Bulky muscle Visible abdominal scar	Large composite defects involving tendon and soft tissue When strong extensor force is required	Yes	Moderate (abdominal weakness, hernia/bulge, scar)	Good functional recovery; reliable strength for knee extension	Abdominal wall dehiscence/hernia Seroma Infection Fat necrosis Partial flap loss
**Gracilis**	Minimal donor-site morbidity Long, reliable vascular pedicle Technically simpler harvest Useful for smaller defects	Small muscle bulk Limited strength compared with other options May not provide sufficient power for large defects	Small to moderate defects Patients with comorbidities Salvage or adjunct procedure	Yes	Minimal (usually well tolerated)	Moderate functional recovery; suitable for low-demand patients or as adjunct	Partial flap loss/necrosis Limited strength gain Scar problems (rare)
**Vastus lateralis**	Biomechanical match to native quadriceps Similar fiber orientation and contraction pattern Good excursion No need for distant donor site	Requires preserved ipsilateral muscle Risk of donor-site weakness Limited availability in bilateral or extensive injury	Quadriceps loss with preserved ipsilateral vastus lateralis Traumatic or oncologic reconstructions	Usually no (pedicle preservation techniques)	Low to moderate (temporary extensor weakness)	Good recovery of active extension; biomechanically advantageous	Temporary donor-site weakness Partial flap loss Hematoma Infection

## Data Availability

No new data were created or analyzed in this study.
